# Radar-based human activity recognition with adaptive thresholding towards resource constrained platforms

**DOI:** 10.1038/s41598-023-30631-x

**Published:** 2023-03-01

**Authors:** Zhenghui Li, Julien Le Kernec, Qammer Abbasi, Francesco Fioranelli, Shufan Yang, Olivier Romain

**Affiliations:** 1 Communication, Sensing and Imaging Group, James Watt School of Engineering, University of Glasgow, Glasgow, G12 8QQ UK; 2 MS3 Group, Department of Microelectronics, Delft University of Technology, Delft, The Netherlands; 3 School of Computing, Edinburgh Napier University, Edinburgh, EH11 4BN UK; 4 ETIS Lab, CY University, Cergy-Pontoise, France

**Keywords:** Engineering, Electrical and electronic engineering

## Abstract

Radar systems are increasingly being employed in healthcare applications for human activity recognition due to their advantages in terms of privacy, contactless sensing, and insensitivity to lighting conditions. The proposed classification algorithms are however often complex, focusing on a single domain of radar, and requiring significant computational resources that prevent their deployment in embedded platforms which often have limited memory and computational resources. To address this issue, we present an adaptive magnitude thresholding approach for highlighting the region of interest in the multi-domain micro-Doppler signatures. The region of interest is beneficial to extract salient features, meanwhile it ensures the simplicity of calculations with less computational cost. The results for the proposed approach show an accuracy of up to 93.1% for six activities, outperforming state-of-the-art deep learning methods on the same dataset with an over tenfold reduction in both training time and memory footprint, and a twofold reduction in inference time compared to a series of deep learning implementations. These results can help bridge the gap toward embedded platform deployment.

## Introduction

Ambient Assisted Living (AAL) aims to provide appropriate healthcare for the increasingly aging population worldwide^1^. It is challenging to support the management of chronic conditions and provide timely assistance for non-communicable diseases (NCD), such as stroke episodes or other anomalies in the patterns of daily activities that may be a sign of deteriorating health. The critical detection of such events at home and the possibility of raising prompt alarms are essential to increase the quality of life of the older and more frail citizens, especially those living in isolation^2^.

In recent years, different sensing technologies have been considered for automatic human activity recognition (HAR), including but not limited to wearable sensors, video-based systems, ambient sensors, and radio frequency (RF) sensors. Radar does not record optical images or videos easily interpretable with the naked eye, which is a benefit in terms of privacy and security in case the information is leaked, or the system is hacked. Furthermore, its contactless sensing capabilities allow monitoring without the patient needing to wear, carry, or interact with sensors.

Radar information in HAR can be presented in multiple domains, including but not limited to range-time, Doppler-time, and range-Doppler. Doppler-time domain or micro-Doppler (mD) signatures are typically used to exploit the small modulations in the received radar signal caused by relative motions of limbs with respect to the trunk^3–5^. Numerous studies in the literature have investigated the use of radar for human activity classification^6–13^. The majority of works have focused on creating and optimizing feature extraction algorithms that generate salient features (e.g., physical, mathematical, and/or textural) that improve the performance for specific applications^7^. However, most of radar based HAR research focuses on spectrograms, i.e., the amplitude of micro-Doppler signatures, whereas other domains are seldom used. Radar data can be represented in a wide range of formats in addition to spectrograms. Finding the optimal radar data domains, as well as the most suitable combination of salient features for a given classification problem becomes an intractable problem.

More recently, deep learning and related classification techniques have gained considerable interest in radar based HAR^8,9,14–18^ as they automatically extract salient features from the radar signatures. However, deep learning methods require a large amount of training data, which is less easy to gather experimentally for radar systems than for other sensing modalities. Furthermore, radar data processing may have high computational cost because of the pre-processing steps of raw data, making it challenging to process in real-time, especially if multiple radar sensors are involved. While general-purpose compute engines, especially graphics processing units (GPUs), have been the mainstay for much processing, less work is done on investigating non-tensor-based computation on resource constraint platforms.

Real-world platforms, such as mobile embedded systems, are inevitably constrained by the hardware. The consideration of the balance between efficiency and performance has emerged when exploring the most suitable algorithms. This aspect of real-time implementation of radar based HAR approaches in constrained platforms has attracted increasing attention, as the natural yet crucial step after classification algorithms development. A real-time end-to-end data-driven model^19^ for through-the-wall HAR can output classification results instantly as the activity happens. Wang^20^ devised an ‘m-Activity’ real-time model to collect and recognize human activities. This model could reduce the noise in the collected data, which addressed the problem of noisy data collection.

Although various solutions have been developed for radar-based human activity classification in indoor scenarios, some important research questions are still not fully answered. First, most current approaches would require a long latency even at the inference/testing stage, because of complex data processing methods or deep neural networks. These research works did not consider the computational cost, focusing on classification accuracy only, so that the results were satisfactory but not always suitable for embedded platforms. It is paramount for realistic deployment to focus on decreasing the footprint of the algorithms in terms of energy consumption as well as on silicon to drive the price of the product for the end-users down. Moreover, many works tend to apply the same algorithm (e.g., using the same feature) to recognize all activities in a multi-class problem, i.e., there are few attempts to capitalize on the diversity of information that can be recorded by various feature combinations and different radar domains.

Expanding on our preliminary results^21^, we propose an adaptive thresholding pre-processing method to focus on the region of interest (ROI) for classification based on patented innovations^22,23^. This approach is designed to reduce the computational load by outlining the ROI, i.e., the most relevant part of a spectrogram also named ‘*mask*’. Afterwards, these ‘masks’ are also applied to the phase, unwrapped phase, and magnitude of the mD signature to highlight the ROI in those domains. A series of specifically designed features for the adaptive thresholding method is also introduced. To increase accuracy and reduce computational loading concurrently, we investigate feature selection and information fusion techniques to optimize performances.

Specifically, compared with our previous paper^21^, this work considers and investigates two new domains of radar information, namely phase and unwrapped phase, which are seldom considered in the literature. Moreover, we expand the implementation of our feature extraction algorithm to new domains, which was not considered in our previous study. In addition, we present a detailed analysis of the effect of the thresholding value selection. Since our new experiments involves a series of new features from different domains, a hierarchical classification model, which divides the standard classification into several stages, is introduced to improve the overall performance by combining different features and domains for each stage. A comprehensive comparison between our methods and other popular neural network-based approaches is also shown.

To summarize, the specific contributions which distinguish this work from the current state of the art are summarized here:

- A novel pre-processing method with adaptive thresholding is proposed for radar based HAR which automatically generates ROI from human mD signatures, with a set of specifically designed features for classification on different domains.
- A comprehensive evaluation of the effect of this adaptive thresholding method on the classification accuracy of individual activities and overall accuracy for the data domains under consideration (mask, masked spectrogram, masked phase, masked unwrapped phase) is provided.
- The optimization of the performance is further analyzed with the fusion of data domains and selection strategies, the use of different parameters of the support vector machine, and the usage of a hierarchical method. These optimizations prove to be very beneficial to boost performances.
- The method is benchmarked against deep learning methods using the same dataset, considering metrics of training time, inference time, model size, number of parameters, accuracy, and memory footprint. This comparison shows that the proposed method can outperform deep learning methods while being computationally efficient and reduce the memory footprint.

## Methods

### Data collection and pre-processing

In this paper, the University of Glasgow Radar Signature dataset^24,25^ was used. The data was collected using an off-the-shelf Frequency Modulated Continuous Wave (FMCW) radar that operates at 5.8 GHz, with a 1 ms pulse repetition period, 400 MHz bandwidth, and 128 complex samples per sweep. Two Yagi antennas were connected to the radar for transmitting and receiving the signals, with a gain of ~  + 17 dBi. A total number of 1754 motion captures were recorded from 72 participants aged 21 to 98 years old. This dataset comprises six types of daily human activities, including walking, sitting, standing, picking up an object, drinking and falling. Note that the dataset is not completely balanced, as the older individuals did not participate in the ‘falling’ activity recording for obvious safety concerns. Table 1 summarizes the details of this dataset.

**Table 1:** Summary of the dataset activities.

No.	Activity description	Number of samples	Data length
A1	Walking back and forth	312	10 s
A2	Sitting down on a chair	312	5 s
A3	Standing up from a chair	311	5 s
A4	Picking up an object	311	5 s
A5	Drinking water	310	5 s
A6	Falling	198	5 s

**Figure 1:**
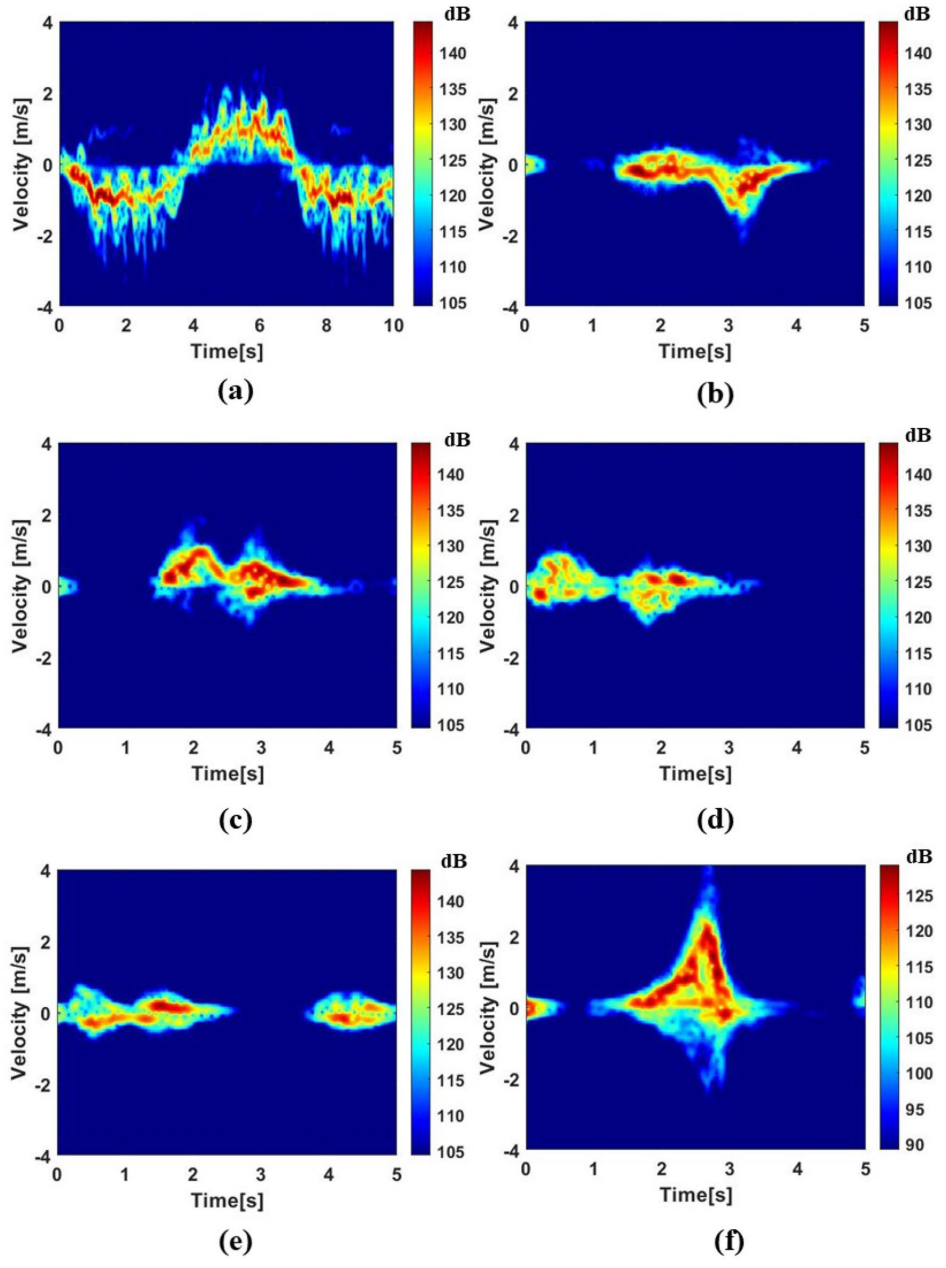
The micro-Doppler signatures of typical samples of the dataset. (**a**–**f**) represent activities A1 ~ A6 micro-Doppler spectrogram.

The following signal pre-processing steps were used to convert the raw data into spectrograms. First, a Hamming-windowed Fast Fourier Transform (FFT) was applied to each pulse, turning them into the range-time map, as well as a 4th-order high-pass Butterworth filter with cut-off frequencies of 0.0075 Hz to remove static clutter. Note that the recording time varies between 5 and 10 s for different data samples, with the number of chirps N = 5000 or N = 10,000, respectively. After acquiring the range-time map, the micro-Doppler signature was generated using a Short-Time Fourier Transform (STFT) on all range bins containing target signatures in the range-time map, utilizing a 0.2 s Hamming window with a 95% overlapping factor. Each sample of A1 activity is divided into two 5 s pieces to ensure its duration is the same as the other activities. Figure 1 depicts the typical spectrogram of each type of activity.

### Adaptive thresholding methods

The aim of the proposed adaptive thresholding approach is to focus only on an ROI containing the contribution of the moving targets in spectrograms for subsequent classification. Areas of the spectrogram that do not convey salient information, such as the portion with low energy (dark blue in the chosen color scale) in Fig. 1, should be discarded.

From Fig. 1 and the samples in the database, we can observe that the intensity varies depending on the activities being performed and the individual performing the activities. This means that it is suboptimal to apply a fixed threshold for all samples as shown in Guo et al.^26^. An adaptive thresholding method is necessary to extract the ROI of each spectrogram.

The proposed technique^21^ uses a specific threshold $T$ to binarize the grayscale mD signature image. This approach focuses on the ROI adaptively by selecting a threshold and then updating it based on the information contained in the window being processed. First, the spectrogram image is transformed into a grayscale image. Suppose that the grayscale image $S$ contains $N$ pixels, and the value of each pixel is represented as $I (x, y)$. Then the initial threshold $\mu$ is defined as in Eq. (1).

1 $$\mu = \frac{1}{N}\sum_{I(x,y)\in S}I(x,y)$$

The grayscale spectrogram image is separated into two portions based on the initial threshold value $\mu$: $P1$ and $P2$, where $P1$ is the image area with a pixel value greater than $\mu$ and P2 is the image area that has a pixel value less than $\mu$. Then, a new threshold $T$ can be determined as in Eq. (2).

2 $$T=\frac{1}{2}\left[\frac{1}{{N}_{1}}{\sum }_{I(x,y)\in {p}_{1}}I(x,y)+\frac{1}{{N}_{2}}{\sum }_{I(x,y)\in {p}_{2}}I(x,y)\right]$$

where $N1$ and $N2$ are the number of pixels in part $P1$ and part $P2$, respectively.

After both $\mu$ and $T$ are obtained, their difference will be compared to a specific parameter: $V$, which can range from 0.05 to 1. According to our previous results^21^, $V = 0.1$ provides satisfactory results, and thus we chose this value for this paper. If the difference is greater than $V$, then $T$ will replace $\mu$ to segment the grayscale spectrogram image and a new $T$ will be calculated using Eq. (2). This process is repeatedly performed until the difference is smaller than $V$, preserving as much of the ROI as possible. The final $T$ value is implemented to binarize the grayscale spectrogram image, as shown in Eq. (3).

3 $$b\left(x,y\right)=\left\{\begin{array}{c}1, I\left(x,y\right)\ge T\\ 0, I\left(x,y\right)<T\end{array}\right.$$

where $b\left(x,y\right)$ is the pixel value of the mask.

**Figure 2:**
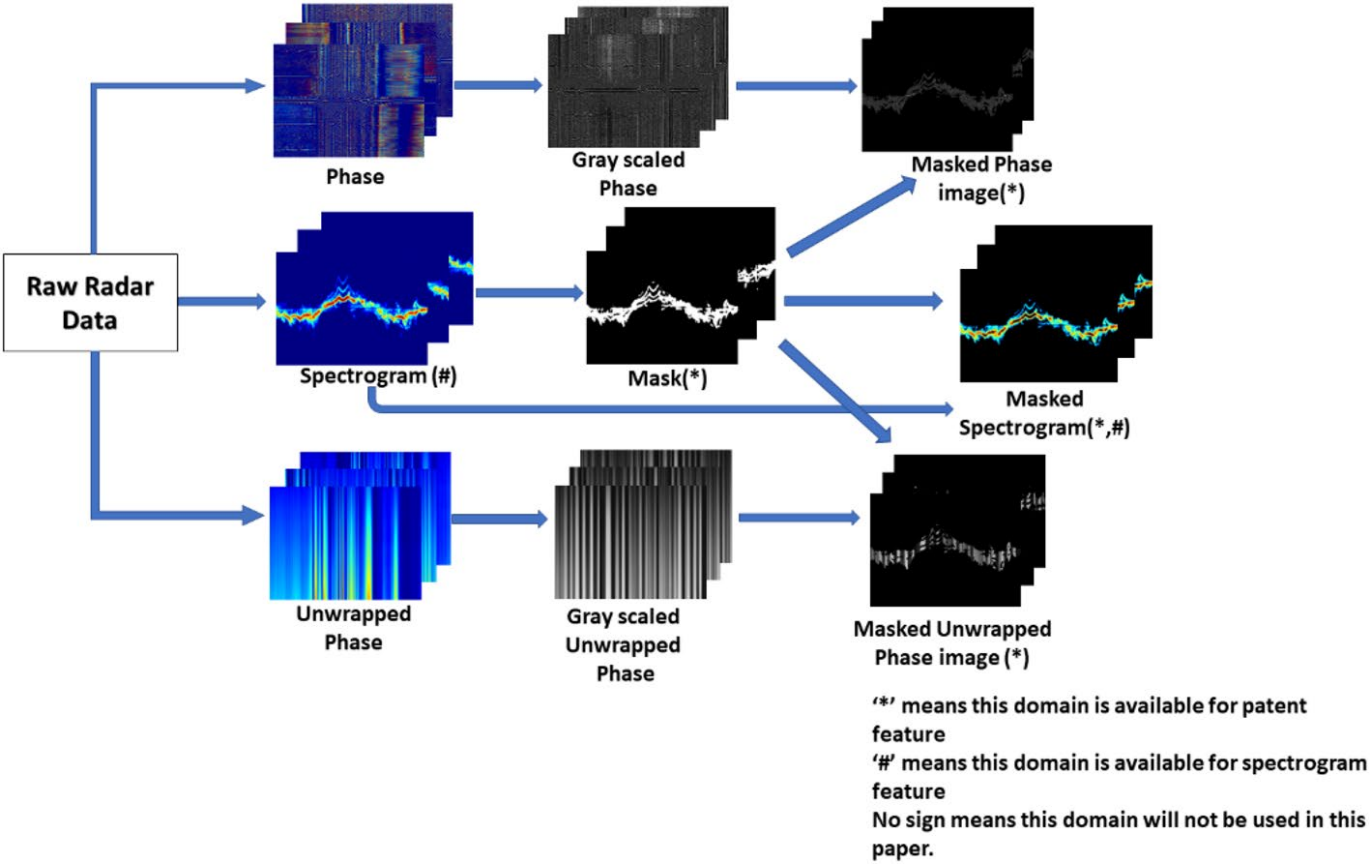
An example of calculating the binary mask to generate masked phase, masked unwrapped phase, and masked spectrogram.

**Figure 3:**
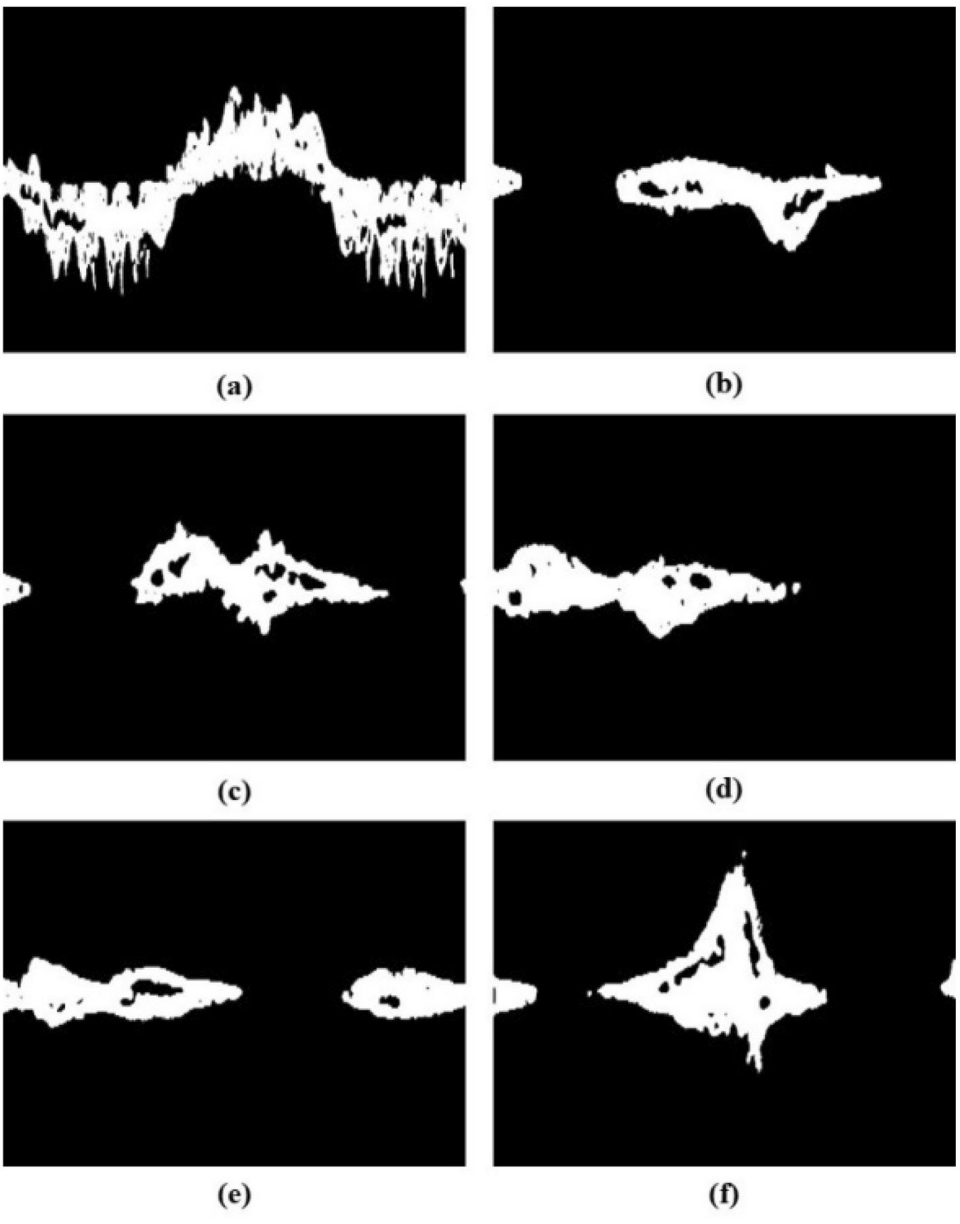
The binary mask of typical samples of the dataset. (**a**–**f**) represent activities A1 ~ A6 micro-Doppler spectrograms.

The binarized image, called ‘mask’, can be used for feature extraction. A mask is applied for this reason on the magnitude, phase, and unwrapped phase of the spectrogram, which are named ‘masked spectrogram’ (amplitude), ‘masked phase’, and ‘masked unwrapped phase’ images, respectively. The process of acquiring the binary mask and masked information is shown in Fig. 2, and the ‘Mask’ samples for each activity are shown in Fig. 3.

### Feature processing and hierarchical structure

The features used in this paper are divided into two groups: group 1 features, also known as ‘patent’ features, due to their correspondence with our patents^22,23^, whereas group 2 features are the ‘radar’ features^7,12,25,27^, which will be referred to as ‘radar’ features in the following section.

- Group 1 (‘patent’) features: 68 features are evaluated, of which two categories are considered: the properties of the ROI and the texture of the image^22,23^. The first category captures the geometrical properties of the ROI, such as centroid, perimeter, and area. The second category is characterized by the spatial distribution of intensity levels within a neighborhood of pixels, which contains information on the spatial arrangements of intensities in an ROI. All the features calculated in this experiment are listed in Table 2.
- Group 2 (‘radar’) features: different types of features are suggested for the spectrograms and masked spectrograms inspired from the previous literature^7,12,25^and from our preliminary results^27^. This includes in total of 21 features, and they are listed in Table 3.

**Table 2:** 68 patent features and their data domains.

	Feature dimensions	Applicable domains
ROI features		
Perimeter of ROI	1 × 1	Mask Masked phase Masked unwrapped Phase Masked spectrogram
Area of ROI	1 × 1	
Centroid of ROI	1 × 2	
Eccentricity of ROI	1 × 1	
Orientation of ROI	1 × 1	
Major and minor axis length of ROI	1 × 2	
Textural features		
Local binary pattern of image	1 × 59	
Moment of image	1 × 1	
The number of total features	68

**Table 3:** 21 radar features and their data domains.

Radar spectrogram features	Feature dimensions	Applicable domains
Entropy of spectrogram	1 × 1	Spectrogram Masked spectrogram
Skewness of spectrogram	1 × 1	
Centroid of spectrogram (mean and variance)	1 × 2	
Bandwidth of spectrogram (mean and variance)	1 × 2	
Energy curve (mean and variance and Trapezoidal numerical integration)	1 × 3	
Singular vector decomposition (mean and variance of the first three vectors of components)	1 × 12	
The number of total features	21	

Note that the data domains of these two groups of features are also listed in Tables 2 and 3.

Feature selection approaches are applied to further improve the performance and reduce the computational complexity^23^. There are mainly three distinct strategies that could be employed: wrapper method, filter method, and embedded method^28^. In this case, we evaluate a wrapper method—sequential floating forward selection (SFFS), which is based on sequential forward selection (SFS). SFS determines the optimal feature combinations by ranking the features in accordance with a classifier and its accuracy as a measure. Unlike the more traditional SFS, SFFS not only adds features progressively, but also eliminates features from the selected subset when the classifier deems it to improve performances after eliminating a specific feature.

Information fusion, the advanced methods for overcoming the limitation of features of a specific domain by combining information or decisions from various sources, comprises in this context. It could be attained through different levels of abstraction^29^, which are commonly divided into three levels—signal, feature, and decision. In this study, both feature level and decision level fusions are used. Feature level fusion cascades the same-labelled features from various sources, as in Eq. (4), where ∩ represents the concatenation of features from different domains.

4 $${F}_{fusion}= {F}_{mask}\cap {F}_{phase}\cap {F}_{unwrap}$$

Decision level fusion merges the classification results from different classifiers into a single outcome. As a classifier, a Naïve-Bayes (NB) combiner^30^ is proposed in this article for the decision level fusion. The mathematical representation of the NB combiner is represented in Eq. (5)^21,30^.

5 $$F({S}_{i})=P({S}_{i}) {\prod }_{m=1}^{N}{p}_{m,k,{S}_{i}}$$

$F({S}_{i})$ indicates the decision factor of class ${S}_{i}$, where ${S}_{i}$ is the class of interest. $F({S}_{i})$ is the product of the support rate $P({S}_{i})$ and the accuracy value of classification confusion matrix entry ${p}_{m,k,{S}_{i}}$ (classifier $m$, row $k$, column ${S}_{1}$). In this experiment, ${S}_{1}$ and k are positive integers ranging from 1 to 6 (6 types of labels in total), $P({S}_{i})$ represents the support rate of the class of interest. For example, suppose that there are 6 classifiers, and 2 of them classify one sample as class ${S}_{1}$, then the support rate $P({S}_{1})$ of this sample is 1/3. ${p}_{m,{S}_{i},k}$ denotes the $({k,S}_{i})$ entry in the confusion matrix for the classifier $m$. The outcome of the fusion will correspond to the class of interest with the highest decision factor.

**Figure 4:**
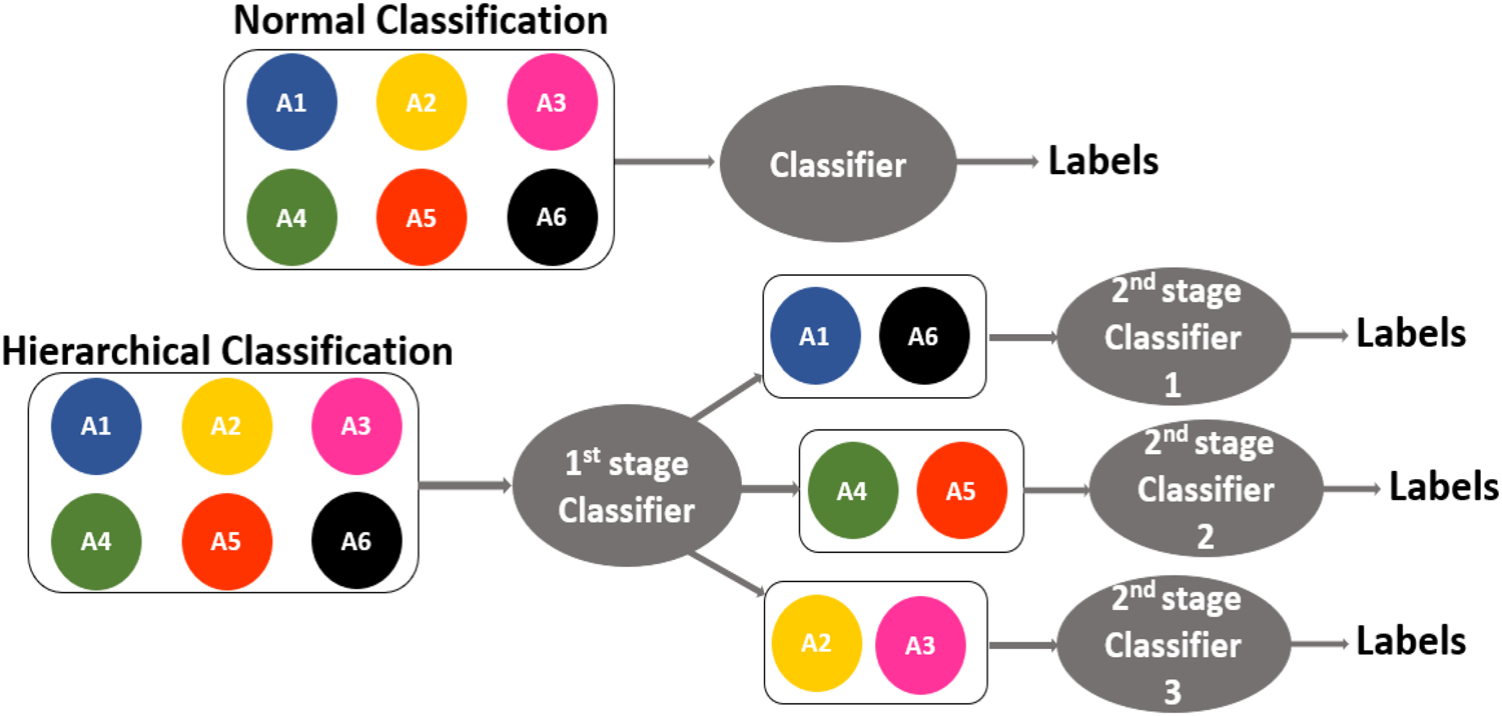
Example of conventional and hierarchical classification.

Unlike traditional supervised classification approaches, which feed all activities into the classifier simultaneously, the proposed hierarchical structure classifies the activities into several sub-groups based on their similarity or misclassification rate. As is shown in Fig. 4 the hierarchical model permits the use of distinct feature sets and algorithms at different stages, and therefore improves the overall performance^31^.

## Results

We begin with an evaluation of the proposed threshold-based approach on spectrograms, phase images and unwrapped phase images, followed by the extraction of features and comparisons among different feature domains. Then, information fusion and feature selection are utilized to improve performances. Finally, we design a hierarchical classification structure based on the prior results to boost the overall performances.

### Human activity classification

Based on the features listed in Tables 2 and 3, the classification models are trained using several support vector machine (SVM) classifiers. SVM is one of the machine learning methods which can be used for the classification task, proposed by Vapnik in the early 1990s^32^. SVM can provide a unique hyperplane to separate learning samples for different classes. This process depends on the choices of kernel functions and hyper-parameters. To analyze which kernel function would be suitable for our data, different kernel functions, namely linear, polynomial (quadratic and cubic), and radial basis kernel (RBF), are implemented and compared with a tenfold cross-validation method. The accuracy is measured as shown in Eq. (6), and the results are given in Table 4. Note that the reported accuracy is averaged over 10 folds.

**Table 4:** Classifications accuracy in % for different SVM kernel functions and different data domains.

%	Linear	Polynomial (quadratic)	Polynomial (cubic)	RBF
Mask	81.0	84.9	83.0	79.9
Masked phase	82.3	83.1	80.6	80.9
Masked unwrapped phase	72.9	73.1	71.1	72.0
Spectrogram	78.0	**80.3**	79.5	78.2
Masked spectrogram (patent)	83.4	**83.6**	80.7	80.0
Masked spectrogram (Radar)	84.1	**85.7**	82.3	84.1
Average across data domains	80.3	81.8	79.5	79.2

6 $$ACC= \frac{True \, Positive+True \, Negative}{Total \, number \, of \, data}$$

According to these preliminary results in Table 4, the SVM model with the quadratic kernel (second-degree polynomial) achieves the highest accuracy consistently across all domains. Furthermore, when comparing the spectrogram to the masked spectrogram (Table 4), the usage of our proposed adaptive thresholding method improves the overall accuracy by 3.3% (from 80.3% to 83.6%) and 5.4% (from 80.3% to 85.7%) when ‘patent’ and ‘radar’ features are used, respectively. Afterwards, an analysis of thresholding values is conducted to further improve the prediction performance, as well as to achieve a better understanding of the interactions between thresholding values, various domains, and the data.

### Threshold values evaluation

To investigate the impact of the adaptive threshold $T$, seven values ranging from $T-10$ to $T+20$ to obtain the binary masks are applied on the spectrogram, phase, and unwrapped phase data. These different data domains are analyzed separately to determine their contribution to classification. They are divided into three types in terms of features: for binary mask and masked (unwrapped) phase, the ‘patent’ features are implemented. For the spectrogram, the ‘radar’ features are used, and for the masked spectrogram data, both ‘patent’ and ‘radar’ features are implemented. At this stage, a robust quadratic-kernel support vector machine (Q-SVM) algorithm with tenfold cross-validation is adopted for activity classification.

Tables 5, 6 and 7 illustrate the initial results using the mask, masked phase, and masked unwrapped phase data domains, with different threshold values. Table 5 shows that an average accuracy of 85.0% is achieved when the binary mask is used with threshold $T-5$. The result of the masked unwrapped image has ~ 10% performance degradation compared with mask and masked phase images. It is mainly because the accuracy decreases greatly in both A2 and A3 activities and slightly in A4 and A5 activities, which are reduced by approximately 20%, 26%, 10% and 5%, respectively. Different thresholds yield the best accuracy for individual activities. For instance, 100% accuracy is achieved for walking with $T+20$ in the masked phase domain.

**Table 5:** Classification results for the mask data domain, with patent features and different threshold values.

%	A1	A2	A3	A4	A5	A6	Avg
T − 10	96.8	91.3	87.7	63.0	77.1	89.9	84.3
T − 5	97.2	92.1	**90.4**	63.4	**77.6**	89.3	**85.0**
T	97.8	90.7	89.3	62.5	76.7	**92.4**	84.9
T + 5	**98.7**	**93.3**	86.9	**64.7**	69.3	89.9	83.8
T + 10	96.1	89.7	90.0	61.2	74.8	90.4	83.7
T + 15	97.4	90.4	90.3	64.4	75.1	91.2	84.8
T + 20	97.8	91.0	88.4	62.0	74.5	90.4	84.0
Avg	97.4	91.2	89.0	63.0	75.1	90.5

**Table 6:** Classification results for the masked phase data domain, with patent features and different threshold values.

%	A1	A2	A3	A4	A5	A6	Avg
T − 10	98.4	88.8	85.8	65.7	70.3	92.6	83.6
T − 5	99.4	86.6	**87.4**	**68.0**	**71.6**	91.9	**84.1**
T	99.7	87.8	86.7	63.4	69.6	91.4	83.1
T + 5	98.4	**89.1**	84.5	61.5	67.8	91.9	82.2
T + 10	99.7	87.1	84.8	61.8	67.1	93.9	82.4
T + 15	99.7	87.5	84.2	59.9	65.1	93.4	81.6
T + 20	**100**	86.2	85.1	59.5	69.3	**94.4**	82.4
Avg	99.3	87.6	85.5	62.9	68.7	92.8

**Table 7:** Classification results for the masked unwrapped phase data domain, with patent features and different threshold values.

%	A1	A2	A3	A4	A5	A6	Avg
T − 10	98.4	67.6	**65.5**	50.2	**70.3**	85.4	72.9
T − 5	**99.4**	69.2	65.1	**54.7**	66.1	85.4	**73.3**
T	98.0	70.1	59.2	54.0	69.3	**87.9**	73.1
T + 5	98.4	65.7	61.6	53.0	68.7	86.6	72.3
T + 10	98.4	**71.1**	61.9	50.2	67.7	86.9	72.7
T + 15	98.7	67.3	59.6	50.5	68.7	87.4	72.0
T + 20	99.4	68.9	60.3	53.0	69.3	84.9	72.6
Avg	98.7	68.5	61.8	52.2	68.6	86.3	

Tables 8, 9 and 10 show the initial results using spectrogram and masked spectrogram data domains, with thresholding range from $T-10$ to $T+20$. Spectrograms with both patent and radar features achieve the highest accuracy at 90.0% with the threshold value $T$. Compared to using both ‘patent’ and ‘radar' features together, implementing only one of them has a negative effect on performances causing a ~ 5% drop in accuracy. For spectrograms with ‘radar’ features and both ‘radar’ and ‘patent’ features, the maximum average accuracy is obtained with threshold $T$ unaltered, which are 85.7% and 90.0%, respectively. The spectrogram with ‘patent’ features reaches its peak accuracy of 84.8% with a threshold value of $T+5$. Comparing the use of ‘radar’ and ‘patent’ features separately on spectrograms, ‘radar’ features yield better performances with ~ 1% improvement overall. However, it should be noted that the ‘patent’ features can be applied on all data domains and not just on mD signatures, so they are in a sense more versatile.

**Table 8:** Classification results for the spectrogram (‘No Mask’) and masked spectrogram data domains, with radar features and different threshold values.

%	A1	A2	A3	A4	A5	A6	Avg
No mask	94.0	*80.2*	82.4	63.6	73.7	87.9	80.3
T − 10	100	87.1	86.3	61.2	70.8	93.1	83.1
T − 5	100	89.3	87.9	62.6	72.1	92.4	84.1
T	**100**	**91.3**	**89.2**	63.9	**75.5**	**94.4**	**85.7**
T + 5	100	89.6	88.4	62.1	71.8	94.4	84.4
T + 10	100	88.0	87.7	61.5	70.3	93.9	83.6
T + 15	99.7	87.1	86.3	63.0	69.6	92.4	83.0
T + 20	99.7	86.7	85.6	**64.4**	67.9	91.7	82.6
Avg	99.9	88.4	87.3	61.7	71.1	93.2

**Table 9:** Classification results for the masked spectrogram data domain, with patent features and different threshold values.

%	A1	A2	A3	A4	A5	A6	Avg
T − 10	100	91.9	**89.1**	62.9	73.8	90.4	84.7
T − 5	99.7	91.6	88.8	61.5	**74.8**	91.9	84.7
T	100	89.8	87	59.8	72.7	92.4	83.6
T + 5	**100**	**91.9**	88.8	61.5	74.1	92.4	**84.8**
T + 10	100	87.1	84.8	61.8	67.1	**93.9**	82.5
T + 15	100	90.9	87.4	**65.4**	73.8	90.4	84.7
T + 20	100	90.5	88.8	60.5	70.6	90.9	83.6
Avg	99.9	90.5	87.8	61.2	72.4	91.8

**Table 10:** Classification results for the masked spectrogram data domain, with both patent and radar features and different threshold values.

%	A1	A2	A3	A4	A5	A6	Avg
T − 10	100	94.7	93.0	**80.1**	78.4	93.4	89.9
T − 5	100	93.7	94.4	79.0	77.3	94.9	89.9
T	**100**	94.7	92.3	77.6	**80.1**	95.4	**90.0**
T + 5	100	**95.1**	90.9	76.6	80.1	95.4	89.7
T + 10	100	93	**93.7**	77.3	79.4	**96.4**	89.9
T + 15	100	91.6	91.2	78.3	79.4	94.4	89.2
T + 20	100	90.4	93.3	75.9	79.0	95.4	89.0
Avg	100	93.3	92.7	77.8	79.1	95.0	

In summary, from this analysis the overall accuracies of mask, masked phase, and masked spectrogram data domains with patent features are increased when the threshold value changes, which means the exploration in this range of threshold values has positive effects on the results. The masked spectrogram with both patent and radar features, outperforms other domains, which has achieved the highest accuracy of 90.0%.

### Feature level fusion and feature selection

After analyzing the performances of the binary mask, masked phase, masked unwrapped phase, and masked spectrogram individually, these data domains are combined with feature level fusion. In each domain, the group with the best overall performance is selected as features for the feature fusion. Based on the previous results, in the fusion for the binary mask and masked phase/unwrapped phase data domains, we only choose the features extracted with threshold $T-5$. For spectrogram and masked spectrogram domains, both features with threshold $T$ are chosen.

At this stage, the Q-SVM and tenfold cross-validation are still used. In this case, we provide seven combinations of features, which are mask + masked phase (Comb 1), mask + masked unwrapped phase (Comb 2), masked phase + masked unwrapped phase (Comb 3), mask + masked spectrogram (Comb 4), mask + masked phase + masked spectrogram (Comb 5), mask + masked unwrapped phase + masked spectrogram (Comb 6) and all together (Comb 7). These are shown in Table 11.

**Table 11:** Classification results for the different combinations of data domains without feature fusion.

%	A1	A2	A3	A4	A5	A6	Avg
Comb 1	99.7	92.3	93.5	80.3	82.3	95.5	90.6
Comb 2	100	88.8	88.2	73.1	82.9	93.4	87.7
Comb 3	98.7	88.8	87.1	67.3	74.5	92.9	84.9
Comb 4	100	**95.4**	**96.1**	78.2	78.2	94.5	90.4
Comb 5	99.7	92.6	93.5	**80.9**	**83.5**	94.2	90.7
Comb 6	98.7	92.3	92.6	79.1	81.2	93	89.9
Comb 7	**100**	95.2	95.0	80.3	80.8	**95.9**	**91.2**

**Figure 5:**
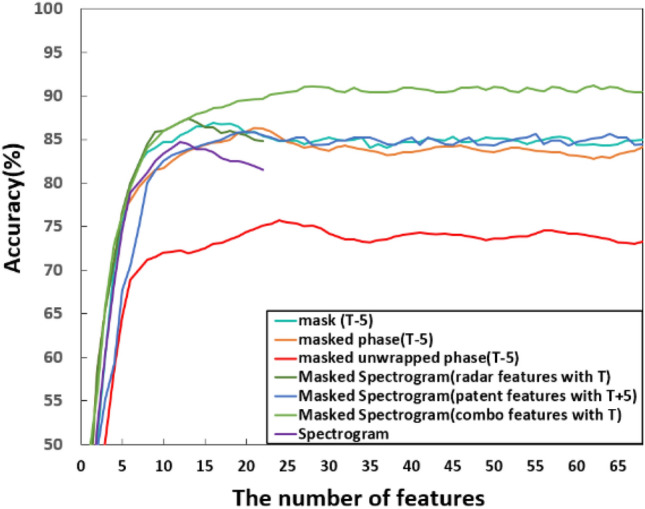
Feature selection with SFFS, results for individual data domains.

**Figure 6:**
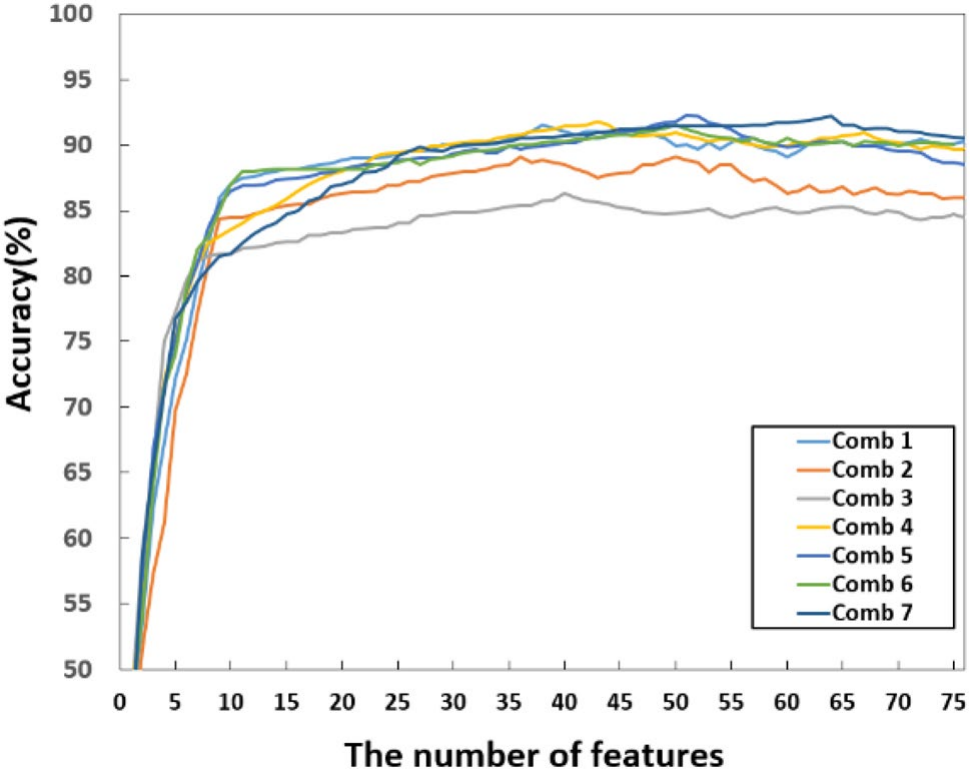
Feature selection with SFFS for feature-level fusion approaches across different data domains.

To further improve the accuracy, reduce the computational load, and evaluate the feature selection approach, the SFFS is applied to both individual results with the best average accuracy and the fusion results of all combos listed above. The individual results are shown in Fig. 5, and the combo results are shown in Fig. 6. These results are also summarized in Table 12.

**Table 12:** Performance comparison using feature selection via SFFS (across different data domains and their combinations).

Methods	No. of original features	No. of selected features	Accuracy (%)
Mask	68	16	86.9
Masked phase	68	21	86.3
Masked unwrapped phase	68	24	75.7
Spectrogram	21	12	84.7
Masked spectrogram (radar features)	21	13	87.4
Masked spectrogram (patent features)	68	20	85.9
Masked spectrogram (both features together)	89	28	91.1
Data domain comb 1	136	38	91.5
Data domain comb 2	136	36	89.1
Data domain comb 3	136	40	86.3
Data domain comb 4	157	43	91.8
Data domain comb 5	225	51	91.3
Data domain comb 6	225	50	91.5
Data domain comb 7	**314**	**64**	**92.2**

The accuracy increase provided by the SFFS is limited. However, the dimension of the feature pool is significantly decreased. Generally, the number of features is reduced by up to ~ 80% compared to the starting count. The accuracy increases by ~ 1% to ~ 4% for individually used data and by ~ 1 to ~ 2% for fusion results.

Note that the binary mask provides the most lightweight implementation with the highest accuracy for individual data domains with 16 features and 86.9%. The masked spectrogram data provides the highest accuracy for single domain use with 91.1% and 28 features. For combined domains, Comb 7 achieves the highest accuracy among all combinations of domains by cascading all types of features, which yields the best accuracy of 92.2% with 64 features. Compared to using single domain features without feature selection, this improvement is from ~ 2% (masked spectrogram) to ~ 18.9% (masked unwrapped phase). However, misclassification events remain, especially for activities A4 and A5.

### Decision level fusion

**Figure 7:**
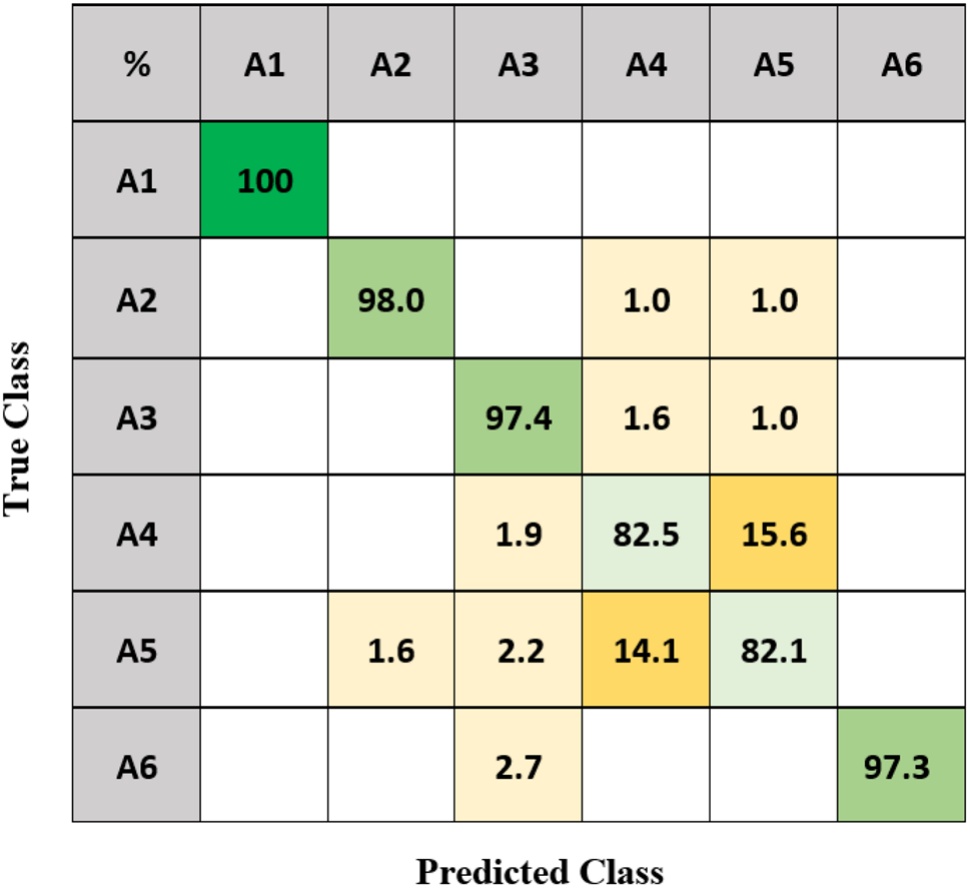
Confusion matrix of Naïve Bayes combiner (decision fusion) with four classifiers.

Based on the previous results, the decision level fusion approach is applied for optimizing classification. Four different approaches, including mask images with threshold $T-5$, masked phase with threshold $T-5$, masked unwrapped phase with threshold $T-5$, and masked spectrogram using combo features with threshold $T$, are combined with NB combiner, since those thresholding values achieved the highest accuracy in their domains (Tables 5, 6, 7 and 10). The confusion matrix of decision level fusion is shown in Fig. 7. The NB combiner outperforms the alternative approaches considered so far with an average accuracy of 92.9%, which improved by + 0.7% compared with the highest accuracy using feature-level fusion (Table 12).

### Hierarchical structure

**Figure 8:**
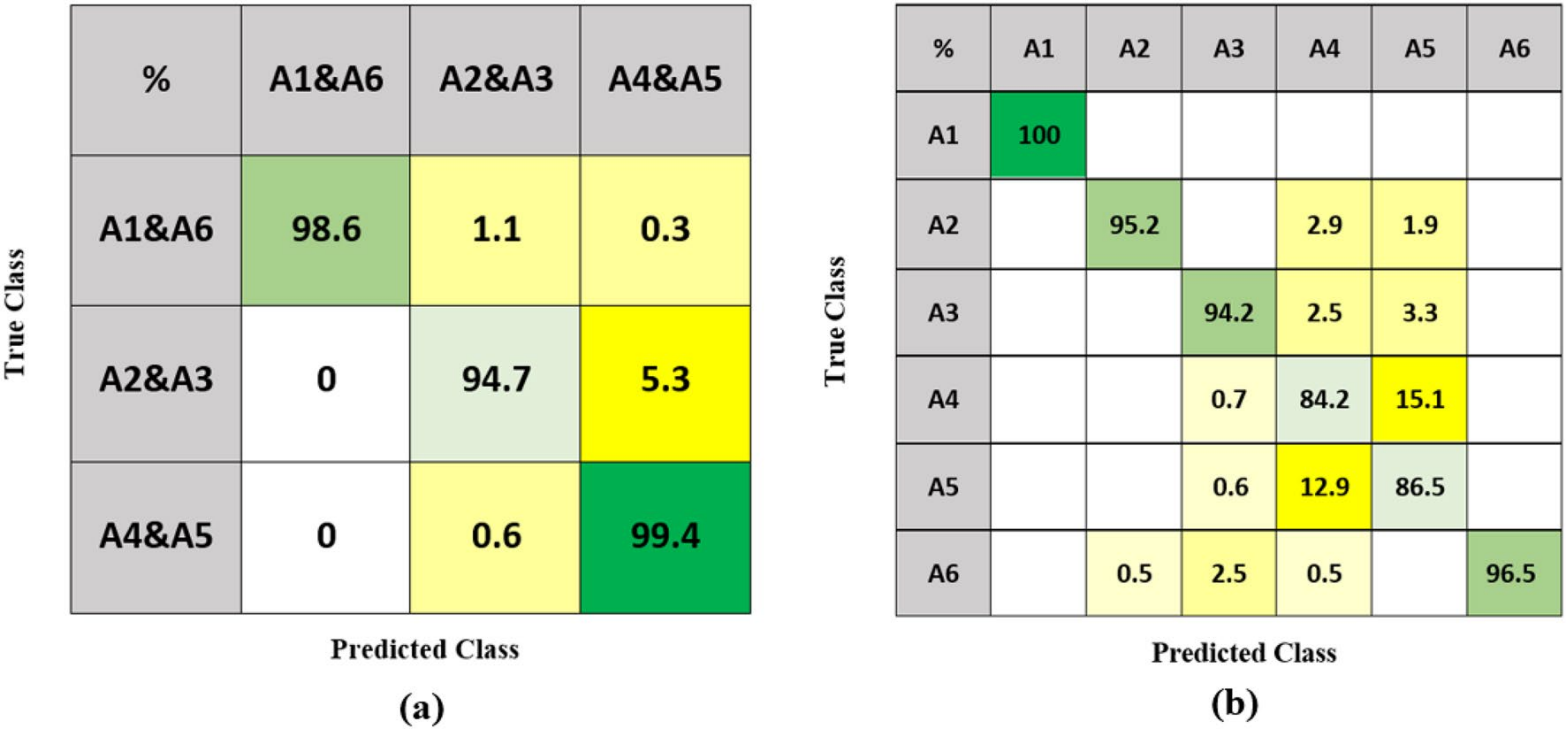
Confusion matrix of 1st stage classification (**a**) and result of the 2nd hierarchical classification (**b**).

The hierarchical structure is applied for optimizing classification. The activities are grouped as in our previous study^27,33^ based on their similarity and false alarm rate. The six activities are first divided into three groups: A1 and A6, A2 and A3, A4 and A5, as shown in Fig. 4. These three pairs will go through the first classification stage, and this is followed for each pair by a binary classification. In the first stage, Comb 7 is implemented with Q-SVM and SFFS (64 features). Comb 7 is also used in the second stage of binary classification for A1&A6. For A2&A3, Comb 4 is used with Q-SVM and SFFS algorithm (43 features). For A4&A5, Comb 5 is applied, with Q-SVM and SFFS (51 features). The confusion matrices of the two classification stages are shown in Fig. 8.

The custom hierarchical structure has an average accuracy of 93.1%, which improved by 0.2% compared with the highest accuracy using decision level classification and 0.9% compared to feature level fusion. Although the accuracies of A2 (− 2.8%), A3 (− 3.2%) and A6 (− 0.8%) decreased by 2.8, 3.2, and 0.8%, respectively, this approach still has the best overall performance. A4 and A5 have the largest improvements with 1.9% and 4.4%, respectively. The accuracy for A1 remains at 100%. A1 consistently has the best performance over the six activities in our experiments. We hypothesize that this is happening because A1, which is walking, is much more diverse than other in-place activities (A2–A6). As a periodic and translational activity, it generates richer Doppler signatures than in-place activities, leading to more distinct features, which make it easier to recognize the activity and thus achieves the best performance.

## Discussion

To evaluate the performance of our methods, different alternative classification models are used with the same dataset including those based on deep learning approaches. The compared models include K-Nearest Neighbor (KNN) model with K = 10, VGG19^34^, Resnet50^35^, NASNet-Mobile^36^, Densenet201^37^, and ShuffleNet^38^. The performance of the models can be analyzed according to three categories—(1) *time*, which refers to how long the model takes to train and to produce an inference; (2) *memory footprint*, which deals with how much memory the model occupies, (3) *accuracy*, which presents the ability to infer the correct class of activities. Specifically, for the time performance, both training time and inference time are assessed separately, and for the memory footprint, the assessment investigates both the number of parameters and the model size. We implement the benchmark analysis on a workstation with an Intel Core I5-9400F CPU 2.9 GHz and NVIDIA GeForce RTX 2060 GPU. The result of this benchmark is shown in Table 13.

**Table 13:** Computational metrics and accuracy comparison of proposed adaptive thresholding method and alternative approaches.

Model	Training time (s)	Inference time (ms)	Model size (MB)	Params (M)	Accuracy (%)	Memory footprint (MB)
KNN	9.25	10.209	1.50	0	85.20	40.21
VGG 19^34^	2173	16.243	558.48	139.60	73.99	2870.69
ResNet50^35^	330	20.111	94.82	23.53	87.93	1468.53
NASNet-Mobile^36^	1889	105.114	19.42	4.28	86.07	1558.85
DenseNet201^37^	2199	87.852	75.08	17.86	91.95	1590.28
ShuffleNet^38^	232	22.267	3.97	1.02	91.02	1435.55
Adaptive thresholding + hierarchical	**20.58**	**15.646**	**2.06**	**0**	**93.10**	**89.13**

The inference time shown in Table 13 is an average per data inference over 30 runs for all models. In general, the time required to train a deep learning model varies depending on the number of network layers. ShuffleNet is the fastest deep learning model in the list, taking 232 s. In comparison to alternative network-based approaches, our approach has the fastest training time of 20.58 s, which is only ~ 9% of the training time of ShuffleNet. VGG-19 is the fastest deep learning method in terms of inference time with 16.243 ms. Our proposed achieves an inference time of 15.646 ms, which is comparable.

The relevant parameters in this analysis are weights that are learnt during training. They are weight matrices that contribute to the model's predictive capability, changed during the back-propagation process. There are millions of parameters produced at the learning stage, and hence the parameters are counted in millions (M). From the comparison of the model sizes in Table 13, we can deduce that the larger the size of the deep learning models, the more parameters they had. The size of VGG19, ResNet50, and DenseNet201 are 558.48 MB, 94.82 MB, and 75.08 MB. On the other hand, NASNet-Mobile and ShuffleNet are much smaller in size at 19.42 MB and 3.97 MB, respectively. The size of our model is only 2.06 MB, which is a 48.11% reduction compared to ShuffleNet and a 99.6% reduction compared to VGG19.

Table 13 also illustrates the accuracy and memory usage of the models using the same dataset which is used in this article. From the memory footprint reported, deep learning models require a considerable memory footprint. ShuffleNet has the lowest footprint in the listed deep learning algorithms. However, our method requires 89.13 MB, which is only 6.21% of the footprint required for ShuffleNet. In addition, our method requires less than one-tenth of the training time compared to the fastest deep learning method while yielding the highest accuracy at 93.10%, which is 1.15% higher than the most accurate deep learning method. Meanwhile, the KNN model with our adaptive thresholding method achieves an accuracy of 85.2%. The result shows that our adaptive thresholding method can also achieve good accuracy with other classifiers instead of SVM, which demonstrates that our method for pre-processing and multi-domain exploration is salient and versatile. This paper proposed a combination of the adaptive thresholding algorithm with the Q-SVM (machine learning based) model, which is more suitable for resource constrained platforms because of its reduced footprint while maintaining speed and increasing accuracy.

## Conclusions

In this paper, we proposed an adaptive thresholding method for radar-based human activity recognition and investigated its performances when applied to spectrogram data for this specific application of HAR. 68 proposed ‘patent’ features are extracted from 4 data domains (mask, masked spectrogram, masked phase, and masked unwrapped phase) and trained with a Q-SVM classifier. The feature level fusion and SFFS selection approach are then used with threshold $T-5$ and masked spectrogram data, with the threshold $T$ that offered the best average accuracy of 92.2% with Comb 7 (combination of mask, masked phase, masked unwrapped phase and masked spectrogram). A further 0.7% improvement was achieved with an NB combiner with decision level fusion reaching 92.9% accuracy. Then, a further improved hierarchical classification structure was proposed to achieve 93.1% accuracy. We have shown that a lightweight implementation of statistical learning combined with efficient pre-processing can outperform deep learning techniques and reduce by over 90% both the memory footprint and the training time bringing us one step closer to implementation on resource-constrained embedded platforms.

For future work, the range of thresholds could be expanded as well as alternative ways of adaptively detecting the ROIs. In the future, we will also explore the relationship between the optimized offset and the radar center frequency and bandwidth. Moreover, since the proposed method aims to operate in real-time conditions, the robustness of the method against noise would need to be investigated. Statistical approaches based on Principal Component Analysis and Canonical Correlation Analysis for features could be used, as well as more radar data domains such as range-time, range Doppler and others^4,7,39,40^ to reduce the handcrafted feature design. For the current dataset, the angular diversity is limited, as this only provides the performing actions in the line-of-sight direction of radar, which is more favourable to collect micro-Doppler signatures. Considering the target angle diversity is important to ensure the robustness of the algorithms with different aspect angles with our seminal work in^4^. We intend to validate experimentally in the coming year or using another public dataset with such data, such as^41^. Also, the dataset currently includes data from adults, which means the performance for children is not considered. Including children can be interesting, especially considering a multitarget scenario (children with adults for example), but this is considered beyond the scope of the reported study and left for future work. The influence of signal processing parameters such as the use of a Hamming window, the length of the range-time data we consider for the spectrogram, the length of the segment of the mD signature from which to extract features as well as the considered parameters could be further optimized. For all these parameters, a global AI-driven approach to tune the signal pre-processing, feature selection, fusion and classification could be investigated. Also, the exploration of evolutionary genetic algorithms could be used to this method for example^42^.

## Data Availability

All data used in the study are available in the manuscript and its tables or online at https://researchdata.gla.ac.uk/848/.
